# Genomic Surveillance and Phylogenetic Analysis of Influenza A(H1N1) pdm09 and A(H3N2) Viruses in Burkina Faso, 2024

**DOI:** 10.1155/av/6901688

**Published:** 2026-08-17

**Authors:** Cathérine Sawadogo, Assana Cissé, Nina Gouba, Abdoul Kader Ilboudo, Madi Savadogo, Moussa Lingani, Benjamin W.O. Kabore, Grissoum Tarnagda, Jean Bienvenue Ouoba, Emilie Dama, Anicet Dahourou, Sandrine Gampini, Hamed Ouedraogo, Zékiba Tarnagda

**Affiliations:** ^1^ National Reference Laboratory for Influenza, Institute for Health Sciences Research, Ouagadougou, Burkina Faso; ^2^ UFR-SVT, Department of Biochemistry-Microbiology, Nazi Boni University, Bobo-Dioulasso, Burkina Faso, univ-bobo.bf; ^3^ Directorate of Animal Health, Ministry of Agriculture, Water, Animal and Fisheries Resources, Ouagadougou, Burkina Faso; ^4^ Clinical Research Unit of Nanoro, Institute for Health Sciences Research, Nanoro, Burkina Faso; ^5^ University Center of Manga, Norbert Zongo University, Manga, Burkina Faso, unz.bf; ^6^ Division of Global Health Protection, U.S. Centers for Disease Control and Prevention, Ouagadougou, Burkina Faso, cdc.gov; ^7^ World Health Organization, Ouagadougou, Burkina Faso, who.int; ^8^ Directorate for Population Health Protection, Ministry of Health, Ouagadougou, Burkina Faso, behdasht.gov.ir; ^9^ One Health Association, Ouagadougou, Burkina Faso

**Keywords:** Burkina Faso, genomic surveillance, H1N1pdm09, H3N2, influenza A, phylogenetics

## Abstract

**Background:**

Influenza is a major cause of acute respiratory infections worldwide. In tropical regions such as Sub‐Saharan Africa, influenza circulates year‐round with irregular peaks, yet genomic data guiding prevention strategies remain limited. This study characterized the genetic diversity and seasonal dynamics of influenza A(H1N1)pdm09 and A(H3N2) viruses circulating in Burkina Faso in 2024.

**Methods:**

A cross‐sectional study was conducted from January to December 2024, including seven sentinel surveillance sites. Patients presenting with influenza‐like illness or severe acute respiratory illness were enrolled. Respiratory specimens were tested by real‐time RT‐PCR. Influenza‐positive samples with a cycle threshold ≤ 30 underwent whole‐genome sequencing using Oxford Nanopore and Illumina platforms. Phylogenetic analyses and clade assignment were performed using MEGA Version 12.

**Results:**

Out of 2951 samples tested, 6.74% were positive for influenza viruses. Females had higher odds of influenza positivity than males (OR = 1.48; 95% CI: 1.11–1.98). A significantly higher risk of positivity was observed in the age groups of 5–15 years (OR = 1.67; 95% CI: 1.07–2.52; *p* = 0.02) and 25–50 years (OR = 2.54; 95% CI: 1.53–4.03; *p* < 0.001). Influenza A(H3N2) peaked in July, while A(H1N1)pdm09 peaked in October. Phylogenetic analysis of 43 genomes revealed co‐circulation of multiple clades within both subtypes.

**Conclusion:**

Influenza A viruses circulating in Burkina Faso in 2024 showed substantial genetic diversity, underscoring the need for continuous genomic surveillance to inform vaccine strain selection and public health strategies in tropical Africa.

## 1. Introduction

Influenza remains a major cause of acute respiratory infections globally, contributing to substantial morbidity and mortality despite the availability of vaccines and antiviral treatments [1], due to the virus’s rapid genetic and antigenic evolution. Each year, the virus infects an estimated 5%–15% of the global population [2–6], resulting in approximately 3–5 million cases of severe disease and 2,90,000–6,50,000 deaths worldwide [2–7].

In Sub‐Saharan Africa, influenza circulation is well documented but characterized by marked spatial and temporal heterogeneity influenced by climatic factors, population mobility, and healthcare access [8, 9]. Such variability underscores the need for context‐specific surveillance data to understand influenza epidemiology in this setting better. Influenza viruses belong to the Orthomyxoviridae family and comprise four types: A, B, C, and D, with Types A and B responsible for most human seasonal epidemics [10–12]. Among circulating influenza A viruses, the A(H1N1)pdm09 and A(H3N2) subtypes are of particular concern due to their rapid antigenic evolution driven by genetic drift, which can undermine vaccine effectiveness and underscores the need for continuous surveillance to inform public health interventions [13].

In Burkina Faso, influenza sentinel surveillance for outpatients presenting with influenza‐like illness (ILI) was established at the National Influenza Reference Laboratory (NIRL) in 2010, following the A(H1N1)pdm09 pandemic [9]. Initially implemented at only two sites located in Bobo‐Dioulasso, the system was progressively expanded to eight sentinel sites distributed across the country’s two largest cities, Bobo‐Dioulasso and Ouagadougou. The surveillance protocol uses WHO/CDC case definitions to identify patients with ILI and severe acute respiratory infection (SARI). Data from pre‐COVID‐19 surveillance indicated regular circulation with peaks during the dry season and early rainy season [14].

However, influenza seasonal patterns were substantially disrupted following the COVID‐19 pandemic, as nonpharmaceutical interventions led to a near‐disappearance of influenza circulation globally during the 2020‐2021 and 2021‐2022 seasons [8, 15, 16]. Following the relaxation of these measures from 2022 onwards, influenza re‐emerged with unusual intensity and atypical seasonal timing [17]. This resurgence has been attributed to the accumulation of an immunity debt within populations insufficiently exposed to influenza viruses during the pandemic period [17]. In Burkina Faso, this postpandemic context may partly account for the modified seasonal dynamics observed in 2023, including an unexpected peak recorded in March [18].

Previous studies in Burkina Faso have documented a substantial burden of influenza. Early reports showed a prevalence of 6.6% among ILI cases between 2010 and 2012 [9]. Subsequent surveillance data from 2014 to 2015 reported higher detection rates, with influenza viruses identified in 15.1% of ILI cases and 6.6% of SARI cases [9, 14]. These studies confirmed the co‐circulation of A(H1N1)pdm09 and seasonal A(H3N2), with A(H3N2) often detected more frequently than A(H1N1)pdm09 [14]. These virus types remain the primary targets of seasonal influenza vaccines for the 2025‐2026 season [19]. However, seasonal influenza vaccine coverage remains extremely limited in Burkina Faso, as seasonal influenza vaccination is not included in the national immunization schedule [20], resulting in near‐absent population coverage. Importantly, our surveillance data confirm an influenza vaccination rate of only 0.7% among the study population [21], reflecting a critical epidemiological vulnerability that directly supports the subsequent discussion on genomic surveillance and potential vaccine mismatch.

Despite the high mutation rate of influenza A viruses and the emergence of new subclades, current vaccines remain reasonably well matched to circulating A(H1N1)pdm09 and influenza B strains, with vaccine effectiveness estimates ranging from 52% to 57% against infection in some regions and up to 75% for preventing influenza‐related hospitalization among children [22]. To support continuous monitoring of viral evolution, early detection of eventual antigenic drift, informed vaccine strain selection, and strengthened pandemic preparedness, the World Health Organization recommends integrating genomic surveillance into routine influenza surveillance [23].

This surveillance is particularly important in West Africa, where multiple influenza lineages co‐circulate, and sporadic introductions linked to international travel are increasingly documented [24]. Sequencing of hemagglutinin (HA) and neuraminidase (NA) genes enables early detection of mutations associated with immune escape, antiviral resistance, and altered receptor binding features of particular relevance for the rapidly evolving A(H1N1)pdm09 and A(H3N2) subtypes [12, 25]. Strengthening genomic surveillance capacity in the region is therefore essential both for regional preparedness and for contributing to global influenza monitoring efforts. Although few studies are carried out on evolutionary dynamics in the region, a 13 year study in Senegal revealed that in 2022, the majority of circulating A(H3N2) strains belonged to Subclade 2a.3a. This subclade differed from the recommended vaccine strain (A/Cambodia/e0826360/2020) due to several amino acid substitutions in the HA gene, such as D69N, N202D, and I208F, which led to a predicted negative vaccine efficacy during that period [26].

Although molecular characterization of influenza A viruses has been documented in Burkina Faso, comprehensive genomic analyses remain lacking.

In this context, the present study aims to provide the first detailed genomic characterization of influenza A(H1N1)pdm09 and A(H3N2) viruses detected in Burkina Faso in 2024, and to describe their seasonal dynamics, phylogenetic relationships, and genetic diversity.

## 2. Materials and Methods

### 2.1. Study Design and Population

A cross‐sectional study was conducted from 1 January to 31 December 2024 by the NIRL (LNR‐G) of the Institute for Research in Health Sciences (IRSS) [18], in collaboration with the Ministry of Health of Burkina Faso. Surveillance and laboratory activities were coordinated by the Directorate for the Protection of Population Health (Direction de la Protection de la Santé des Populations [DPSP]). Seven sentinel surveillance sites were included: the Center for Health and Social Promotion (CSPS) of Bolomakoté, the CSPS of Colsama, the Medical Center with Surgical Unit (CMA) of Hounde, the CMA of Boussé, the CMA of Kongoussi, the National Teaching Hospital of Bogodogo, and the Hospital Saint Camille of Ouagadougou. These sites were distributed across four regions: Kadiogo, Kuilsé, Oubritenga, and Guiriko. Patients presenting with ILI or SARI were enrolled according to WHO case definitions [9, 14].

### 2.2. Sample Collection and Processing

Sample collection and processing were conducted as previously described [27] following the national influenza sentinel surveillance protocol, and standardized case report forms were used (Supporting file 1). Nasopharyngeal and/or oropharyngeal swabs were collected and placed in viral transport medium. Samples were stored at 2°C–8°C and transported to the LNR‐G via the national integrated specimen referral system (SITEB) [28]. Upon receipt, samples were aliquoted and either stored at −80°C or processed within one week [18].

### 2.3. Laboratory Testing and Sequencing

Viral RNA was extracted using the QIAamp Viral RNA Mini Kit (QIAGEN, Hilden). Samples were screened for influenza A, influenza B, and SARS‐CoV‐2 using the CDC Influenza SARS‐CoV‐2 (FluSC2) Multiplex real‐time RT‐PCR assay (CDC, Atlanta) [29]. Samples were considered positive for influenza A if the cycle threshold value for the influenza A gene target was ≤ 40, in accordance with the manufacturer’s instructions. Influenza A‐positive samples were subsequently subtyped using CDC real‐time RT‐PCR assays targeting the HA gene [18].

### 2.4. Full‐Genome Amplification and Library Preparation

Full‐genome amplification was performed using a multisegment RT‐PCR (MS‐RT‐PCR) protocol, applied exclusively to samples with Ct values ≤ 30. This universal amplification method utilizes a single‐tube reaction with conserved primers (MBTuni12/13) targeting the common terminal sequences at the 3′ and 5′ ends of all eight genomic segments, enabling simultaneous full‐length amplification of the entire influenza A viral genome [30, 31]. MS‐RT‐PCR products were purified using the eCube PCR Purification Kit (PhileKorea, Korea). Purified double‐stranded DNA (dsDNA) was quantified using the Qubit 1X dsDNA High Sensitivity Assay Kit (Invitrogen, USA) and normalized to 0.2 ng/μL prior to library preparation.

### 2.5. Sequencing Platforms and Bioinformatics

Sequencing was performed using two platforms as part of a collaborative effort. Oxford Nanopore Technologies libraries were prepared using the Rapid Barcoding Kit 96 V14 (SQK‐NBD114.96) and sequenced on a MinION Mk1C at the National Reference Laboratory for Influenza (LNR‐G) in Burkina Faso, while Illumina sequencing was conducted using the DNA Prep Kit on a MiSeq (2 × 250 bp) platform at the CDC (Atlanta, USA). For bioinformatics analysis, reads were assembled using MIRA for Nanopore reads and IRMA for Illumina data, and only sequences achieving ≥ 80% genome coverage were retained for further analysis. The assembled influenza virus genomes meeting the ≥ 80% genome coverage threshold were submitted to GISAID. The accession numbers assigned to the influenza A(H1N1)pdm09 virus sequences were EPI_ISL_20054665, EPI_ISL_20054664, EPI_ISL_20054663, EPI_ISL_20054662, EPI_ISL_20054661, EPI_ISL_20054614, EPI_ISL_20054613, EPI_ISL_20054612, EPI_ISL_20051754, EPI_ISL_20050821, EPI_ISL_19495408, EPI_ISL_19495407, EPI_ISL_19495406, EPI_ISL_19495405, EPI_ISL_19495402, EPI_ISL_19495400, EPI_ISL_19495399, EPI_ISL_19495397, EPI_ISL_19495393, EPI_ISL_19495389, and EPI_ISL_19495388, while the accession numbers assigned to the influenza A(H3N2) virus sequences were EPI_ISL_20327702, EPI_ISL_20054688, EPI_ISL_19495325, EPI_ISL_19495324, EPI_ISL_19495322, EPI_ISL_19495321, EPI_ISL_19495320, EPI_ISL_19495319, EPI_ISL_19495318, EPI_ISL_19495317, EPI_ISL_19495314, EPI_ISL_19495313, EPI_ISL_19495309, EPI_ISL_19495308, EPI_ISL_19495305, EPI_ISL_19495303, EPI_ISL_19495302, EPI_ISL_19495300, EPI_ISL_19495295, EPI_ISL_19495293, and EPI_ISL_19495290.

### 2.6. Phylogenetic and Statistical Analyses

While whole‐genome sequencing was initially performed on the platforms to generate the data, the subsequent phylogenetic and evolutionary analyses presented in this study were specifically reconstructed using the full‐length HA gene sequences of both influenza A(H1N1)pdm09 and A(H3N2) viruses. In addition to the sequences generated in Burkina Faso, publicly available HA sequences collected during the same period (20232024) from neighboring African countries, including Côte d’Ivoire, Cameroon, Kenya, Niger, Togo, Ghana, and Senegal, were retrieved from the GISAID database to provide regional context. Whenever possible, approximately 10 representative sequences per country were selected; otherwise, all available sequences from the corresponding period were included. To place the Burkina Faso viruses within the broader global evolutionary context, representative Northern Hemisphere reference strains from the 2023‐2024 and 2024‐2025 influenza seasons were incorporated into the dataset. These included A/Victoria/4897/2022 and A/Wisconsin/67/2022 for A(H1N1)pdm09, and A/Massachusetts/18/2022, A/Thailand/8/2022, and A/Darwin/9/2021 for A(H3N2). Appropriate outgroup sequences were also included to improve tree rooting and evolutionary interpretation, namely, A/California/04/2009 for A(H1N1)pdm09 and A/Switzerland/9715293/2013 for A(H3N2). Clade assignment and phylogenetic analyses were performed using MEGA Version 12, with sequence alignments generated through the MUSCLE algorithm. Phylogenetic trees were reconstructed using the maximum likelihood method based on the Kimura 2‐parameter (K2P) model, with 1000 bootstrap replicates to assess node robustness. Epidemiological data were analyzed using *R* software (Version 4.3.2). Univariate analysis was performed to compare categorical variables using chi‐square tests, and odds ratios with 95% confidence intervals were calculated to identify associated factors. A *p* value < 0.05 was considered statistically significant.

### 2.7. Ethical Considerations

This study was conducted as part of routine national influenza surveillance activities. Authorization to use surveillance data was obtained from the Health Research Ethics Committee of Burkina Faso (clearance certificate number: 2025‐473). The data of all patients were anonymized in accordance with the Declaration of Helsinki [32]. Written informed consent was obtained from all participants prior to sample collection (or from parents/legal guardians in the case of children). This consent was documented on the study’s notification form, signed by each participant or their guardian, which included a statement confirming that they had been informed of the sampling and testing procedures and agreed to proceed.

## 3. Results

### 3.1. Study Population and Sociodemographic Characteristics

A total of 2951 patients were enrolled during the study period, of whom 6.74% tested positive for influenza A viruses. Univariate logistic regression analysis revealed several factors significantly associated with influenza positivity. Positivity was significantly higher among females than males (OR = 1.48; 95% CI: 1.11–1.98; *p* = 0.008) (Table 1). Regarding age, a significantly higher risk of positivity was observed in the 5–15 years group (OR = 1.67; 95% CI: 1.07–2.52; *p* = 0.02) and the 25–50 years group (OR = 2.54; 95% CI: 1.53–4.03; *p* < 0.001). The 15–25 years age group did not reach statistical significance, although it bordered the threshold (OR = 1.87; 95% CI: 0.95–3.35; *p* = 0.05) (Table 1). In terms of geographic distribution, using the Kadiogo region as the reference category, significantly lower odds of influenza positivity were observed in the Kuilsé (OR = 0.39; 95% CI: 0.20–0.69; *p* = 0.002) and Guiriko regions (OR = 0.35; 95% CI: 0.25–0.49; *p* < 0.001). In contrast, no statistically significant difference was found for the Oubri region (Table 1).

**TABLE 1:** Sociodemographic and clinical factors associated with influenza A positivity among the study population, univariate analysis, Burkina Faso, 2024.

Variables	*N* test (%)	% positive	OR (crude) (95% CI)	*p* value
Sex				
Male	1498 (50.8%)	5.54	Ref.	
Female	1453 (49.2%)	7.98	1.48 (1.11–1.98)	0.008^∗∗^
Age group				
0–5 years	2274 (77.1%)	5.72	Ref.	
5–15 years	305 (10.3%)	9.18	1.67 (1.07–2.52)	0.02^∗^
15–25 years	118 (4.0%)	10.17	1.87 (0.95–3.35)	0.05
25–50 years	165 (5.6%)	13.33	2.54 (1.53–4.03)	< 0.001^∗∗∗^
50+ years	89 (3.0%)	7.87	1.41 (0.58–2.90)	0.40
ILI/SARI				
ILI	2165 (73.4%)	7.16	Ref.	
SARI	786 (26.6%)	5.60	0.77 (0.54–1.08)	0.14
Health region				
Kadiogo	413 (14.0%)	13.80	Ref.	
Kuilsé	239 (8.1%)	5.86	0.39 (0.20–0.69)	0.002^∗∗^
Guiriko	2216 (75.1%)	5.28	0.35 (0.25–0.49)	< 0.001^∗∗∗^
Oubritenga	83 (2.8%)	13.25	0.95 (0.46–1.84)	0.89
Type of specimen				
Nasopharyngeal swab	2843 (96.3%)	6.58	Ref.	
NP/OP swabs	11 (0.4%)	27.27	5.33 (1.16–18.58)	0.01^∗^
Oropharyngeal swab	97 (3.3%)	9.28	1.45 (0.67–2.78)	0.30

*Note:* Values derived from univariable logistic regression. Ref.: Reference category.

Abbreviations: CI, confidence interval; OR, odds ratio.

^∗^ *p* < 0.05.

^∗∗^ *p* < 0.01.

^∗∗∗^ *p* < 0.001.

### 3.2. Seasonal Dynamics of Influenza and Subtypes

**FIGURE 1:**
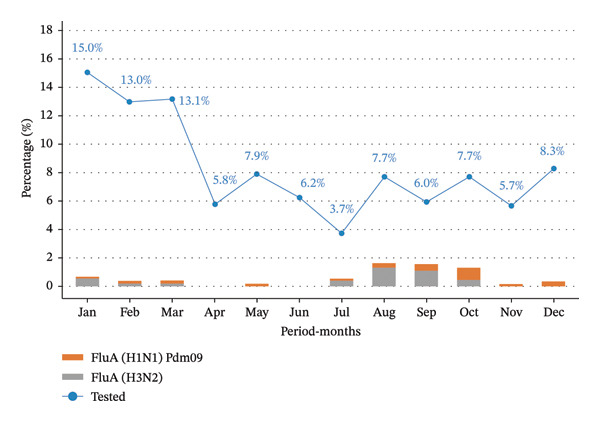
Temporal distribution of acute respiratory infections and influenza virus detection in Burkina Faso, 2024. The blue line represents the monthly proportion of respiratory samples collected and tested for influenza during the surveillance period. The stacked bars represent the proportion of samples positive for influenza A subtypes, including A(H3N2) (gray) and A(H1N1)pdm09 (orange), among the samples tested each month.

Despite monthly variations in the number of samples collected, influenza viruses circulated throughout the year with two distinct periods of increased activity. A moderate wave occurred between January and April, with peaks in January and March, followed by a more pronounced wave from May to November, peaking in July (Figure 1). Influenza A(H3N2) predominated during the mid‐year peak, whereas A(H1N1)pdm09 activity peaked in October. Influenza A(H3N2) and A(H1N1)pdm09 circulated simultaneously throughout the year (Figure 1).

### 3.3. Phylogenetic Analysis

Of the 81 influenza A‐positive samples submitted for sequencing (36 in Atlanta and 45 at the LNR‐G), 43 genomes met quality criteria for analysis, including 18 (22.22%) A(H1N1)pdm09 and 25 A(H3N2) (38.86%) viruses. A(H1N1)pdm09 viruses predominantly belonged to Clade 6B.1A.5a.2a (94.44%). Several amino acid substitutions were identified in antigenic sites of the HA protein. In the Sa site, Substitutions T131K and T135K were observed in multiple isolates; P137S was detected in the Sb site. In the Ca site, Substitutions A186T and Q189E were identified. One isolate in Subclade D.3.1 additionally carried mutations N156K and S162N, which are associated with impaired serum neutralization. The antigenic implications of these substitutions are discussed in the Discussion section.

**FIGURE 2:**
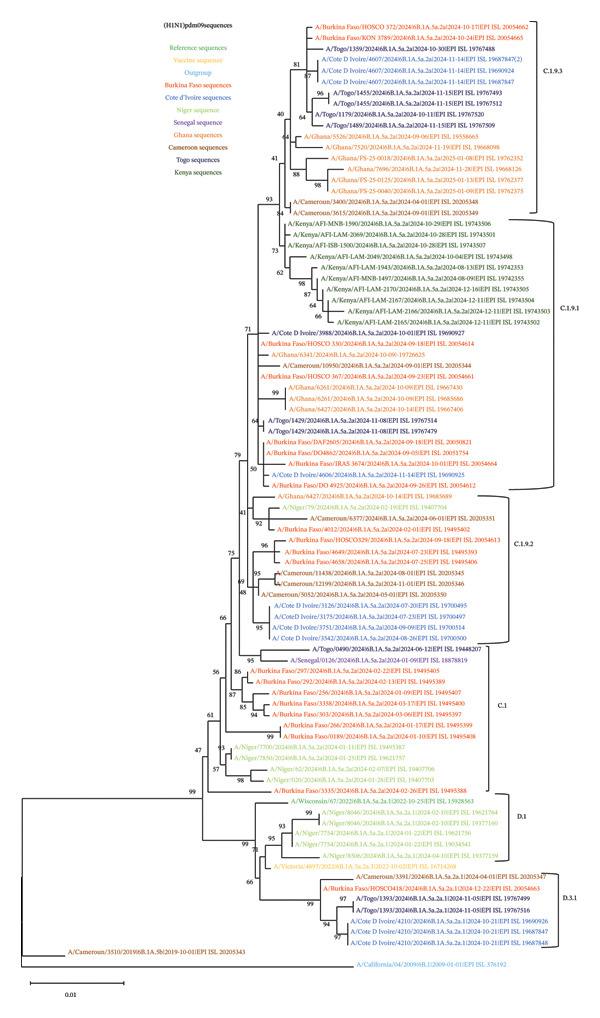
Phylogenetic tree of A(H1N1)pdm09 sequences generated in 2024, reconstructed using the maximum likelihood method based on the Kimura 2‐parameter (K2P) model in MEGA Version 12 with 1000 bootstrap replicates and visualized using FigTree V1.4.4.

Sequences generated from the CDC Atlanta during the first half of 2024 clustered within Subclades C.1, C.1.9, and C.1.9.2, whereas sequences produced at the NIRL were distributed across Subclades C.1.9 and D.3.1 (Figure 2).

### 3.4. Phylogenetic Analysis of H3N2 Viruses

**FIGURE 3:**
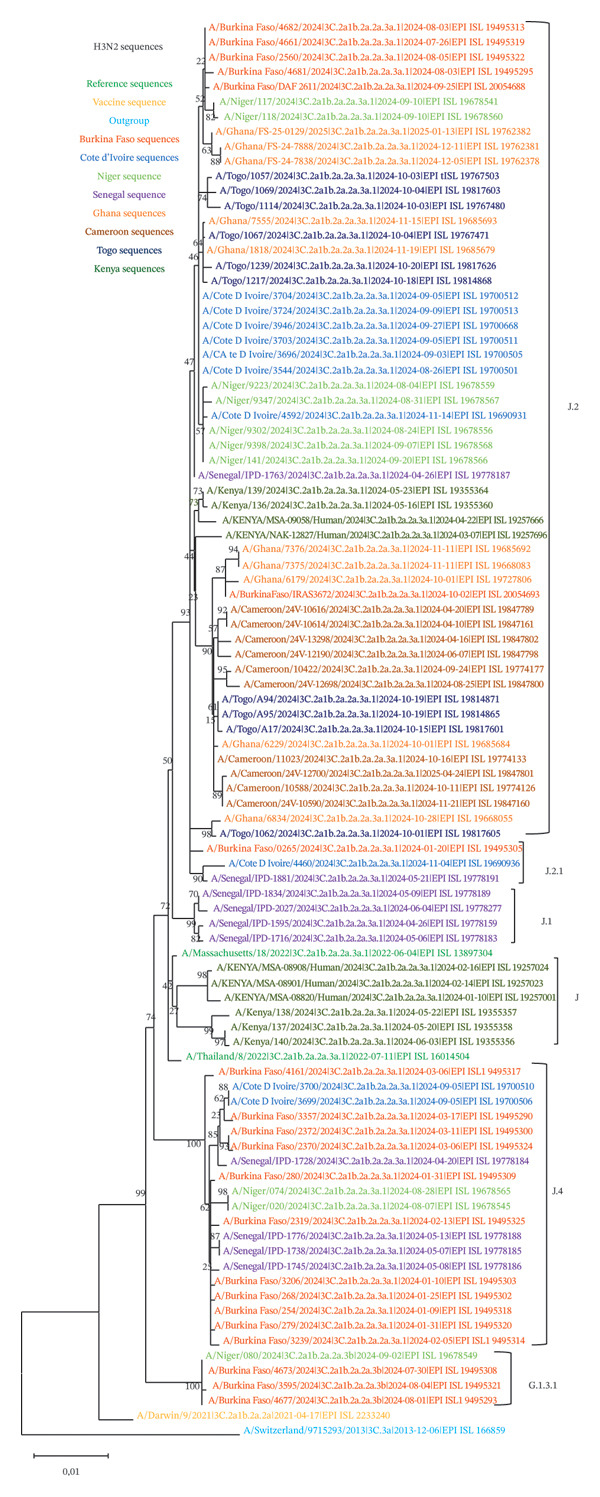
Phylogenetic tree of A(H3N2) sequences generated in 2024, reconstructed using the maximum likelihood method based on the Kimura 2‐parameter (K2P) model in MEGA Version 12 with 1000 bootstrap replicates, and visualized using FigTree V1.4.4.

The A(H3N2) viruses clustered within Clades 3C.2a1b.2a.2a.3b and 3C.2a1b.2a.2a.3a.1, including Subclades G.1.3.1, J.2, and J.4. Sequences generated in Burkina Faso were exclusively assigned to Clade 3C.2a1b.2a.2a.3a.1, Subclade J.2. In contrast, sequences obtained from influenza surveillance specimens collected in Burkina Faso and subsequently characterized by the CDC showed a broader distribution across multiple subclades, including G.1.3.1, J.2, and J.4 (Figure 3). Each A(H3N2) subclade was characterized by distinct molecular signatures in the HA protein. Subclade J.2 viruses carried Substitutions T135K, H156Q, and F193S relative to the A/Darwin/9/2021 vaccine strain. Subclade J.4 viruses exhibited K92R and I192V substitutions. Subclade G.1.3.1 strains retained a mutation profile closer to the 2021 vaccine strain but also harbored additional substitutions reflecting ongoing genetic drift. A detailed summary of amino acid substitutions by subclade is provided in Supporting Information.

This divergence is clearly visible in the phylogenetic tree, as shown by the large horizontal gap between the 2021 baseline and the 2024 top nodes (Figure 3). These new lineages have accumulated numerous mutations, reflecting the ongoing genetic drift.

## 4. Discussion

This study provides the first comprehensive genomic description of influenza A(H1N1)pdm09 and A(H3N2) viruses circulating in Burkina Faso in 2024. Influenza virus activity, assessed by the monthly positivity rate of influenza A viruses among tested sentinel surveillance samples, was characterized by sustained circulation with two seasonal peaks, consistent with patterns observed in other tropical settings [9, 14, 18]. The predominance of A(H3N2) during the major mid‐year peak aligns with observations from neighboring West African countries [33].

Over years of surveillance, influenza in Burkina Faso has shown year‐round circulation with two periods of increased activity [9, 14, 18]. In 2024, a moderate wave occurred between January and April, followed by a more pronounced wave from May to November corresponding to the second seasonal peak [9, 14, 18]. This pattern differs from earlier reports indicating higher activity between January and March and contrasts with findings from other tropical settings such as Senegal, Cambodia, and Côte d’Ivoire, where two more evenly distributed peaks were observed [33–35]. These results suggest a redistribution rather than a loss of influenza seasonality, with a predominance of the second peak. This shift may partly reflect the lasting effects of COVID‐19 public health measures and altered population immunity following the pandemic, as documented in several studies in Sub‐Saharan Africa and other tropical settings [8, 18]. However, since these observations are based on surveillance data alone, a definitive causal relationship cannot be established, and other contributing factors, including changes in healthcare‐seeking behavior, surveillance intensity, and waning population immunity, should also be considered.

The results of this study revealed significant geographic variation in influenza A positivity, with lower odds in the Kuilsé and Guiriko regions compared to the Kadiogo region. This heterogeneity may reflect differences in population density, mobility, and access to healthcare services. The Kadiogo region, which includes the capital Ouagadougou, is characterized by high population density and increased mobility that may facilitate respiratory virus transmission. Similar spatial variations have been reported in West Africa, where influenza transmission differs according to demographic characteristics and surveillance performance [36]. Previous studies in Burkina Faso also reported heterogeneous influenza circulation, particularly in major urban centers such as Ouagadougou and Bobo‐Dioulasso.

Also, age was significantly associated with the risk of infection. Adults aged 25–50 years had higher odds of influenza positivity compared to children under five years. However, this finding should be interpreted with caution as it likely reflects a selection bias rather than true physiological susceptibility. Young children frequently present with ILI caused by a wide array of other pediatric respiratory pathogens (such as respiratory syncytial virus, rhinovirus, and human metapneumovirus), which can dilute the overall influenza positivity rate in this age group. In contrast, adults are rarely tested or seek hospital care for these common cold viruses, meaning adults meeting the ILI criteria are more specifically enriched for true influenza infections. This syndromic overlay represents a known limitation of surveillance based strictly on clinical definitions. Similar trends have been reported in influenza surveillance studies in West Africa, Ghana [37]. These findings highlight the potential role of socially active age groups in influenza transmission and the importance of targeted prevention strategies.

The major challenge for influenza control remains antigenic drift [2, 38, 39]. Our genomic analysis highlights a mismatch between the 2023‐2024 vaccine strains and the viruses circulating in Burkina Faso. For A(H1N1)pdm09, the vaccine strain A/Victoria/4897/2022 belongs to Clade 6B.1A.5a.2a.1. However, 94.44% of our sequences clustered within Clade 6B.1A.5a.2a, while only a single sequence belonged to the Vaccine clade 6B.1A.5a.2a.1.This pattern is consistent with reports from China during the 2022‐2023 influenza season [40] and from Romania during the 2022‐2023 and 2023‐2024 seasons [41], where a clear predominance of Clade 6B.1A.5a.2a was also observed, with only a limited number of sequences assigned to Clade 6B.1A.5a.2a.1. The concordance of these findings across geographically distant regions supports the notion of globally synchronized circulation of A(H1N1)pdm09 viruses and suggests that their recent evolutionary dynamics are largely driven by the international dissemination of dominant lineages rather than by independent local evolution [42].

Several isolates were identified as belonging to Subclades C.1.9 and D.3.1, likely due to substitutions in key HA regions, including the Sa and Ca antigenic sites and the receptor‐binding site [43, 44]. These substitutions are known to reduce vaccine‐induced antibody recognition and include T131K, T135K, P137S, A186T, and Q189E [45]. One of the isolates clustered within Subclade D.3.1, carrying several mutations (N156K, S162N, P137S, A186T, and Q189E) associated with impaired serum neutralization [46]. These changes suggest ongoing drift in the 6B.1A.5a.2a lineage consistent with global reports in 2024 [47, 48]. These findings have important implications for influenza control in Burkina Faso, since they suggest that the current vaccine strains may offer reduced protection against circulating viruses. This antigenic mismatch could compromise vaccine effectiveness and highlights the need for continued monitoring of circulating strains to detect emerging variants in order to guide immunization strategies.

All A(H3N2) viruses detected in Burkina Faso clustered exclusively within Clade 3C.2a1b.2a.2a.3a.1 (Subclade J), which was not included in the 2023 and 2024 vaccine formulation, based on A/Darwin/9/2021 [49]. Worldwide, A(H3N2) viruses evolved toward a distinct subclade (e.g, 2a.3a.1), reducing vaccine effectiveness and prompting its selection for the 2024 and 2025 vaccine [50]. The absence of Clade J.2 in the 2023 and 2024 vaccine suggests limited protection, especially in this region.

Overall, the divergence between circulating viruses and vaccine strains emphasizes the strategic importance of integrated genomic surveillance. Such systems allow real‐time tracking of antigenic drift and inform global vaccine‐strain selection, especially critical in tropical settings with irregular influenza seasonality.

In summary, influenza viruses circulating in Burkina Faso in 2024 showed increasing antigenic diversification, which may have implications for vaccine match based on globally recommended strains.

Seasonal influenza vaccination is not currently part of the national prevention strategy in Burkina Faso, and vaccine use remains very limited. As consideration of influenza vaccination policies expands in low‐ and middle‐income countries, locally generated genomic data are essential to inform evidence‐based decision‐making. Integrating routine genomic surveillance within existing influenza monitoring systems will be important for anticipating viral evolution and strengthening preparedness in tropical settings.

This study has several limitations. Only slightly more than two‐thirds of samples were successfully sequenced due to low viral loads and variable specimen quality, which may affect representativeness. Use of different sequencing platforms may have introduced technical variation. Differences across sentinel sites likely reflect specimen quality, healthcare‐seeking behavior, and geographic context.

## 5. Conclusion

This study provides new information on influenza circulation in Burkina Faso and complements molecular characterization conducted through routine sentinel surveillance for a comprehensive analysis. We document sustained viral activity with distinct seasonal waves and genetic diversity consistent with global patterns. Our findings also demonstrate the country’s growing capacity to generate reliable genomic data, a key asset for strengthening national surveillance systems. Despite limitations, the results highlighted the need to fully integrate genomic sequencing into routine influenza monitoring to improve early detection, outbreak response, and preparedness for future respiratory threats.

## Author Contributions

Cathérine Sawadogo, Assana Cissé, Nina Gouba, and Zékiba Tarnagda conceptualized the study and contributed to the development of the methodology.

Assana Cissé and Cathérine Sawadogo were responsible for data collection and conducted the laboratory analyses.

Cathérine Sawadogo and Abdoul Kader Ilboudo contributed to data curation and statistical analysis. Cathérine Sawadogo drafted the first version of the manuscript. Zékiba Tarnagda, Nina Gouba, Assana Cissé, Abdoul Kader Ilboudo, Moussa Lingani, Emilie Dama, Anicet Dahourou, Hamed Ouedraogo, Madi Savadogo, Jean Bienvenue Ouoba, Grissoum Tarnagda, and Sandrine Gampini critically reviewed and substantially revised the manuscript.

## Funding

This study received no specific funding.

## Disclosure

All authors read and approved the final version of the manuscript before submission and agree to be accountable for its content.

## Conflicts of Interest

The authors declare no conflicts of interest.

## Supporting Information

Additional supporting information can be found online in the Supporting Information section.

## Supporting information


**Supporting Information** Supporting File 1: Standardized case report forms.

## Data Availability

The datasets used and/or analyzed during the current study are available from the corresponding author on reasonable request. All data were anonymized, and no individual‐level identifiable information is presented in this manuscript.
